# Analysis of Risk Factors for Hypoparathyroidism After Total Thyroidectomy

**DOI:** 10.3389/fsurg.2021.668498

**Published:** 2021-05-21

**Authors:** Zhou Ru, Wang Mingliang, Wang Maofei, Chen Qiaofeng, Yuan Jianming

**Affiliations:** Department of General Surgery, School of Medicine, Ruijin Hospital/Lu Wan Branch, Shanghai Jiaotong University, Shanghai, China

**Keywords:** total thyroidectomy, hypoparathyroidism, logistic regression analysis, neck surgery, risk factor

## Abstract

**Objective:** To analyze the risk factors of hypoparathyroidism after total thyroidectomy.

**Methods:** Clinical data of patients who undergo total thyroidectomy in the Luwan Branch of Ruijin Hospital Affiliated to Medical College of Shanghai Jiaotong University was collected from January 2015 to December 2018, retrospectively. Logistic regression was used to analyze the risk factors associated with transient and long-term hypoparathyroidism.

**Results:** A total of 537 patients were collected. The patients' average age included in the study was 47.3 ± 12.7 years old, including 135 males (25.1%) and 702 females (74.9%). There were 194 patients (36.1%) with transient postoperative hypoparathyroidism, and 21 patients (3.9%) had long-term postoperative hypoparathyroidism. After multivariate analysis, the main risk factors related to postoperative transient hypoparathyroidism were gender (*P* = 0.038, OR 0.686), combined lymph node dissection (*P* = 0.008, OR 1.569), and the maximum diameter of the thyroid (*P* = 0.011, OR 1.192), second operation (*P* = 0.001, OR 1.974), preoperative blood calcium (*P* < 0.001, OR 0.028). The main risk factors associated with long-term postoperative hypoparathyroidism are combined with lymph node dissection (*P* = 0.011, OR 1.594), maximum thyroid diameter (*P* = 0.032, OR 1.254), and PTH on the first day after surgery (*P* < 0.001, OR 1.199).

**Conclusions:** Gender, combined lymph node dissection, maximum thyroid diameter, a second surgery, and preoperative blood calcium are risk factors for transient hypoparathyroidism after thyroid surgery. The combined lymphatic dissection and the thyroid gland's maximum diameter are risk factors for long-term hypoparathyroidism after thyroid surgery. PTH on the first day after surgery has a predictive effect on patients with long-term hypoparathyroidism.

## Introduction

Hypoparathyroidism is a common complication after thyroidectomy. It is reported in the literature that its incidence can reach 1.2–40% (1). A small number of patients are converted to long-term hypoparathyroidism. The incidence of persistent hypocalcemia up to 0~5% (2), the main clinical manifestations are reducing parathyroid hormone (PTH), leading to osteoporosis, muscle spasm, etc., which are the main factors affecting the quality of life of patients after thyroid surgery. The purpose of this study is to explore the possible related factors that affect postoperative hypoparathyroidism and provide an objective basis for reducing surgical complications.

## Materials and Methods

### Research Design

Clinical data of patients undergo thyroidectomy in the Luwan Branch of Ruijin Hospital Affiliated to Medical College of Shanghai Jiaotong University was collected from January 2015 to December 2018, retrospectively. Primary criteria for inclusion: Patients undergoing bilateral thyroidectomy for benign and malignant thyroid diseases. Primary criteria for exclusion: (1) Patients with hypocalcemia before surgery (serum Ca < 2.2 mmol/L); (2) Patients with second thyroid surgery; (3) Patients with missing postoperative follow-up data. (4) There are patients with renal insufficiency affecting serum calcium and phosphorus metabolism before surgery. (5) Patients undergoing parathyroid surgery. The data of 537 patients were collected. The average age of patients included in the study was 47.3 (16–77) years old, with 135 males (25.1%), and 402 females (74.9%). All data are collected from the medical history system and entered by one person, and the other person is responsible for data verification. Demographic data, surgery-related data, and perioperative inspection indicators were mainly collected.

### Judgment and Treatment of Postoperative Hypoparathyroidism

Routine preoperative examinations were performed to take venous blood to test blood calcium and parathyroid hormone and retest in the morning the next day after surgery. Serum calcium lower than 2.2 mmol/L or parathyroid hormone lower than 7 pg/ml is defined as transient hypoparathyroidism after surgery, and oral calcium supplements and vitamin D3 are given. Re-testing of the parathyroid hormone at 6 months after surgery is still <7 pg/ml. Supplementation of calcium and vitamin D3 is required to maintain normal blood calcium (≥2.2 mmol/L) is defined as long-term hypoparathyroidism (3).

### Statistical Methods

SPSS 13.0 software (SPSS Inc., Chicago, IL) was used for statistical analysis. The count data were presented in percentage; the normal distribution data in the measurement data was presented in the mean ± standard deviation. The non-normal distribution data was presented in the form of it is presented in the form of digits (range). The measurement data adopt the Independent Samples *T*-test, and the enumeration data adopt the chi-square test and Fisher exact probability. Multivariable analysis was done by Logistic regression, Enter method, to evaluate the risk factors of transient and long-term hypoparathyroidism. *P* < 0.05 was considered statistically different.

## Result

### Demographic

Five hundred and thirty-seven patients undergoing thyroid surgery were included, 135 males (25.1%) and 402 females (74.9%), aged 47.3 ± 12.7 years.

### Perioperative Data

The operation time for all patients was 94.1 ± 37.9 min. All subjects collected postoperative pathological data, 42 cases (7.8%) were benign, and 495 cases (92.2%) were malignant. Among the malignant tumors, there were 489 cases of papillary carcinoma (91.1%), 4 cases of follicular carcinoma (0.007%), and two cases of medullary carcinoma (0.004%). One hundred and thirty-seven cases (25.5%) underwent simple gland resection, and 400 cases (74.5%) combined with lymphatic dissection (Table 1).

**Table 1:** Baseline data and perioperative data of all patients.

		**Hypoparathyroidism group**	**Unhypoparathyroidism group**	***P*-value**	***X2/t***
*N* (case)		194	343		
Gender (cases)	Male (%)	37 (27.4)	98 (72.6)	0.022	5.94
	Female (%)	157 (39.1)	245 (61.9)		
Age (year)		47.7 ± 12.9	47.1 ± 13.0	0.708	−0.121
Operation time (min)		102.0 ± 36.6	89.6 ± 34.6	<0.001	−4.762
Type (case)	Benign	13 (31.0)	29 (79.0)	0.413	0.53
	Malignant	181 (36.6)	314 (73.4)		
Combined lymphatic dissection (case)	No (%)	22 (16.1)	115 (83.9)	<0.001	32.10
	Yes (%)	172 (43.0)	228 (67.0)		
Tumor diameter (mm)		18.8 ± 14.0	17.8 ± 12.7	0.296	1.046
Thyroid diameter (cm)		4.89 ± 1.34	4.41 ± 1.09	0.001	−3.457
Second operation (case)	Yes	15 (62.5)	9 (37.5)	0.006	7.33
	No	181 (35.3)	332 (64.7)		
Preoperative blood calcium (mmol/L)	2.38 ± 0.07	2.42 ± 0.08	<0.001	3.961	
Preoperative PTH (ng/L)	35.5 ± 17.6	35.3 ± 15.4	0.888	−0.139	

The average preoperative blood calcium of all patients was 2.40 ± 0.08mmol/L, the average parathyroid hormone was 35.4 ± 16.1 pg/L, the average blood calcium of the next day after surgery was 2.23 ± 0.12 mmol/L, and the average parathyroid hormone was 15.8 ± 11.3 pg/L. Postoperative transient hypoparathyroidism occurred in 194 cases (36.1%), among which 135 cases (25.1%) had parathyroid hormone lower than normal, and 156 cases (29.1%) had blood calcium lower than normal. Long-term hypoparathyroidism occurred in 21 cases (3.9%), of which 19 cases had parathyroid hormone lower than normal, and two cases had normal parathyroid hormone but required calcium supplementation to maintain normal blood calcium.

### Results of Multivariate Logistic Regression Analysis

Multivariate analysis showed that gender, combined lymph node dissection, maximum thyroid diameter, a second surgery, preoperative blood calcium, and transient hypoparathyroidism were related (*P* < 0.05). The OR value of gender (male) is 0.686, 95% CI is (0.535, 0.805); the OR value of combined lymphatic dissection is 2.974, 95% CI is (2.155, 4.103); the OR value of whether lymph node dissection is 1.569, 95% CI It was (1.122, 2.194); the OR value of the maximum thyroid diameter was 1.192, 95% CI was (1.041, 1.364); the OR value of preoperative blood calcium was 0.028, 95% CI was (0.004, 0.203) (Table 2).

**Table 2:** Multivariate logistic regression analysis results of transient hypoparathyroidism.

**Factor**	***P*-value**	**OR (95%CI)**
Gender (male)	0.038	0.686 (0.535, 0.805)
Age (year)	0.881	1.001 (0.989, 1.013)
Combined lymphatic dissection	0.008	1.569 (1.122, 2.194)
Operation time (min)	0.064	1.005 (1.000, 1.009)
Tumor diameter (mm)	0.139	0.991 (0.978, 1.003)
Thyroid diameter (cm)	0.011	1.192 (1.041, 1.364)
Second operation (yes)	0.001	1.974 (1.155, 3.103)
Preoperative blood calcium (mmol/L)	<0.001	0.028 (0.004, 0.203)
Preoperative PTH (ng/L)	0.382	0.996 (0.987, 1.005)

*Assignment description*.

Combined lymphadenectomy, the maximum diameter of the thyroid gland, and PTH on the first day after surgery (d1PTH) were associated with long-term hypoparathyroidism (*P* < 0.05). The OR value of combined lymphadenectomy was 1.594, 95% CI was (0.586, 4.334); The OR value of Maximum thyroid diameter was 1.254, 95% CI was (0.794, 1.981), the OR value of d1PTH was 1.199, and 95% CI was (1.086, 1.324) (Table 3).

**Table 3:** Multivariate logistic regression analysis results of long-term hypoparathyroidism.

**Factor**	***P*-value**	**OR (95%CI)**
Gender (male)	0.568	0.717 (0.229, 2.246)
Age (year)	0.469	0.986 (0.950, 1.024)
Combined lymphatic dissection	0.011	1.594 (0.586, 4.334)
Operation time (min)	0.071	0.976 (0.956, 0.996)
Tumor diameter (mm)	0.076	0.956 (0.909, 1.005)
Thyroid diameter (cm)	0.032	1.254 (0.794, 1.981)
Second operation (yes)	0.131	1.764 (1.132, 3.251)
Preoperative blood calcium (mmol/L)	0.816	0.481 (0.001, 228.086)
Preoperative PTH (ng/L)	0.060	0.965 (0.930, 1.001)
d1 Blood calcium (mmol/L)	0.376	4.376 (0.167,114.361)
d1 PTH (ng/L)	<0.01	1.199 (1.086,1.324)

## Discussion

Hypoparathyroidism is one of the most common complications after thyroid surgery. Surgery affects the parathyroid glands' blood supply, and direct damage to the parathyroid glands or miscut during the operation are the most common causes. In this study, the incidence of transient hypoparathyroidism was 36.1%, and the incidence of hypoparathyroidism was 3.9% after 6 months of follow-up. In 537 patients undergoing bilateral total thyroidectomy, the following conclusions were obtained after multivariate logistic regression analysis: gender, combined lymphatic dissection, operation duration, maximum thyroid diameter, preoperative blood calcium, and transient hypoparathyroidism are related, Combined with lymphatic dissection, maximum thyroid diameter, and d1PTH are associated with long-term hypoparathyroidism.

Malignant thyroid tumors often require lymph node dissection. Related literature also shows that lymph node dissection in the central group can significantly increase the direct damage to the parathyroid glands or blood supply disorders during the operation (4). Patients underwent preventive neck lymph node dissection, and there is considerable controversy in domestic and foreign related literature. Some scholars believe that preventive dissection does not significantly reduce the probability of recurrence and metastasis and increases the risk of parathyroid injury. This case study suggests that combined lymph node dissection is a risk factor for transient and long-term hypoparathyroidism, which is similar to the results of some studies at home and abroad (5). Therefore, to reduce the damage to the parathyroid glands during lymph node dissection, it is necessary to perform lymph node dissection under the premise of standardized protection of the parathyroid glands and use nano carbon or other color reagents to color the parathyroid glands (6).

Regarding the size of the thyroid and the second operation, it is believed that the increase in the size of the thyroid and the difficulty of neck anatomy will cause the reduction of the surgical field and the difficulty of identifying and protecting the parathyroid glands (7). Therefore, for complicated operations, more attention should be paid to identifying, and protecting parathyroid glands. In univariate analysis, operation time was related to transient postoperative parathyroid hypoplasia, but it was excluded in multivariate analysis. It should be explained that the difficulty of the operation played a leading role, leading to prolonged operation time.

Preoperative blood calcium is the baseline of the patient's blood calcium level. This study used blood calcium (more objective) and PTH laboratory test indicators to determine hypoparathyroidism. In order to rule out some subjective factors, the main complaint of patients-related hypocalcemia symptoms was not used. Therefore, the preoperative blood calcium index has a direct effect on whether the postoperative calcium is low. But this study also found that it does not correlate with long-term postoperative parathyroid hypoplasia. D1PTH impacts long-term hypoparathyroidism, and it can be used as a predictor of long-term hypoparathyroidism. For patients whose d1PTH is less than normal, calcium and vitamin D3 supplementation should be strengthened. If failing to get enough calcium supplements or close follow-up review, the patient may miss the chance of parathyroid function recovery.

## Conclusion

Hypoparathyroidism after bilateral total thyroidectomy is common, and most of them are transient. Gender, combined lymphatic dissection, maximum thyroid diameter, a second surgery, and preoperative blood calcium are the risk factors for transient hypoparathyroidism after thyroid surgery. A small number of transient hypoparathyroidisms turned into long-term hypoparathyroidism. The combination of lymphatic dissection and the largest thyroid diameter were risk factors for long-term hypoparathyroidism after surgery. The d1PTH was lower than Normal has a predictive effect on patients with long-term hypoparathyroidism.

## Data Availability Statement

The raw data supporting the conclusions of this article will be made available by the authors, without undue reservation.

## Ethics Statement

Ethical review and approval was not required for the study on human participants in accordance with the local legislation and institutional requirements. The patients/participants provided their written informed consent to participate in this study.

## Author Contributions

ZR, YJ, and WMi contributed to the conception and design of the study. WMa organized the database. YJ performed the statistical analysis and wrote the first draft of the manuscript. ZR wrote sections of the manuscript. All authors contributed to manuscript revision, read, and approved the submitted version.

## Conflict of Interest

The authors declare that the research was conducted in the absence of any commercial or financial relationships that could be construed as a potential conflict of interest.

## References

[1] Dionigi G Baeuzzi A Bertocchi V Carrafiello G Boni L Rovera F. Prospectives and surgical usefulness of perioperative parathyroid hormone assay in thyroid surgery. Expert Rev Med Devices. (2008) 5:699–704. 10.1586/17434440.5.6.699 19025346

[2] Toniato A Boschin IM Piotto A Pelizzo M Sartori P. Thyroidectomy and para-thyroid hormone: tracing hypocalcemia-prone. Am J Surg. (2008) 196:285–8. 10.1016/j.amjsurg.2007.06.036 18466858

[3] Shoback D. Clinical practice. Hypoparathyroidism. N Engl J Med. (2008) 359:391–403. 10.1056/NEJMcp0803050 18650515

[4] Landry CS Grubbs EG Hernandez M Hu MI Hansen MO Lee JE. Predictable criteria for selective, rather than routine, calcium supplementation following thyroidectomy. Arch Surg. (2012) 147:338. 10.1001/archsurg.2011.1406 22184134 PMC4809199

[5] Su AP Wang P Gong YP Gong R Li Z Zhu J. Risk factors of hypoparathyroidism following total thyroidectomy with central lymph node dissection. Medicine. (2017) 96:e8162. 10.1097/MD.0000000000008162 28953664 PMC5626307

[6] Yin FY Xing H Zhang XJ Zhang S Yang G. Protective effects of nano-carbon on parathyroid in thyroid surgery. Chin J Endocr Surg. (2015). 2:144–6. 10.3760/CMA.J.ISSN.1674-6090.2015.02.015

[7] Zheng JW Song HM Cai SY Wang Y Han X Wu H. Evaluation of clinical significance and risk factors of incidental parathyroidectomy due tothyroidectomy: a single-center retrospective clinical study. Medicine. (2017) 96:e8175. 10.1097/MD.0000000000008175 28953673 PMC5626316

